# Migraine, Celiac Disease and Intestinal Microbiota

**DOI:** 10.15844/pedneurbriefs-31-2-3

**Published:** 2017-02

**Authors:** Hakim Rahmoune, Nada Boutrid

**Affiliations:** 1 Pediatrics Department, Setif University Hospital; Setif-1 University, Algeria; 2 Genetic & Nutritional Diseases Laboratory; Setif-1 University, Algeria

**Keywords:** Celiac Disease, Gastrointestinal Microbiota, Headache

## Abstract

Investigators from four European tertiary care hospitals (in Paris, France; Milan, Udine and Perugia, Italy) performed a case-control study of children and adolescents aged 6 to 17 years diagnosed with primary headaches in the emergency department by a pediatric neurologist using the validated ICHD-3 criteria.

Investigators from four European tertiary care hospitals (in Paris, France; Milan, Udine and Perugia, Italy) performed a case-control study of children and adolescents aged 6 to 17 years diagnosed with primary headaches in the emergency department by a pediatric neurologist using the validated ICHD-3 criteria. They enrolled 648 controls and 424 cases (257 patients with migraine and 167 with tension-type headache). Investigators masked to a patient’s group allocation diagnosed functional gastrointestinal disorders using the Rome III diagnostic criteria. Eighty-three (32%) children and adolescents in the migraine group were diagnosed with functional gastrointestinal disorders compared with 118 (18%) in the control group (p<0·0001). Multivariable logistic regression showed a significant association between migraine and three gastrointestinal disorders: functional dyspepsia, irritable bowel syndrome and abdominal migraine. The authors concluded that correct recognition would have an impact on the diagnosis and therapeutic management of these pediatric gastrointestinal disorders. [1]

COMMENTARY. This well conducted multicenter trial included all functional gastrointestinal disorders defined according to ROME III criteria. However, among digestive afflictions, celiac disease (CD) deserves particular attention. CD patients may exhibit a myriad of extra-intestinal symptoms, which includes neurological symptoms such as migraine. Studies report a high frequency of migraines in patients with CD and vice versa, and describe the beneficial effect of a gluten-free diet in these cases [2]. Also, the prevalence of CD among children with irritable bowel syndrome (IBS) is reported to be 4 times higher than among the general pediatric population [3]. Using the Rome III diagnostic criteria of functional gastrointestinal disorders would help to distinguish CD from the broad pool of IBS, with a potential consequent relief of migraine upon initiation of the appropriate diet.

On the other hand, recent findings regarding the role of the gastrointestinal microbiota in the gut-brain axis suggests that an unbalanced gut flora (i.e. dysbiosis) can be associated with neurological diseases like migraine. Enhanced pro-inflammatory immune responses have been reported with intestinal disorders associated with dysbiosis and increased intestinal permeability (just like IBS and celiac disease) as well as in migraine patients [4]. Evidence suggests that alterations in gut microbiota could be a potent mediator in migraine [5]; this might explain, at least partly, the current study results. We are, definitely, what we eat!

## References

[1] Le Gal J, Michel JF, Rinaldi VE, Spiri D, Moretti R, Bettati D (2016). Association between functional gastrointestinal disorders and migraine in children and adolescents: a case-control study. The Lancet Gastroenterology & Hepatology.

[2] Mormile R (2014). Celiac disease and migraine: is there a common backstage?. Int J Colorectal Dis.

[3] Cristofori F, Fontana C, Magistà A, Capriati T, Indrio F, Castellaneta S (2014). Increased prevalence of celiac disease among pediatric patients with irritable bowel syndrome: a 6-year prospective cohort study. JAMA Pediatr.

[4] van Hemert S, Breedveld AC, Rovers JM, Vermeiden JP, Witteman BJ, Smits MG (2014). Migraine associated with gastrointestinal disorders: review of the literature and clinical implications. Front Neurol.

[5] Hindiyeh N, Aurora SK (2015). What the Gut Can Teach Us About Migraine. Curr Pain Headache Rep.

